# A POLE Splice Site Deletion Detected in a Patient with Biclonal CLL and Prostate Cancer: A Case Report

**DOI:** 10.3390/ijms22179410

**Published:** 2021-08-30

**Authors:** Markus Steiner, Franz J. Gassner, Thomas Parigger, Daniel Neureiter, Alexander Egle, Roland Geisberger, Richard Greil, Nadja Zaborsky

**Affiliations:** 1 Department of Internal Medicine III with Haematology, Medical Oncology, Haemostaseology Infectiology and Rheumatology, Oncologic Center, Salzburg Cancer Research Institute—Laboratory for Immunological and Molecular Cancer Research (LIMCR), Cancer Cluster Salzburg, Paracelsus Medical University, 5020 Salzburg, Austria; mark.steiner@salk.at (M.S.); f.gassner@salk.at (F.J.G.); t.parigger@salk.at (T.P.); a.egle@salk.at (A.E.); r.geisberger@salk.at (R.G.); r.greil@salk.at (R.G.); 2 Department of Biosciences, Paris-Lodron-University Salzburg, 5020 Salzburg, Austria; 3 Institute of Pathology, Cancer Cluster Salzburg, Paracelsus Medical University, 5020 Salzburg, Austria; d.neureiter@salk.at

**Keywords:** chronic lymphocytic leukemia, prostate cancer, case report, immunoglobulin light chain, POLE

## Abstract

Chronic lymphocytic leukemia (CLL) is considered a clonal B cell malignancy. Sporadically, CLL cases with multiple productive heavy and light-chain rearrangements were detected, thus leading to a bi- or oligoclonal CLL disease with leukemic cells originating either from different B cells or otherwise descending from secondary immunoglobulin rearrangement events. This suggests a potential role of clonal hematopoiesis or germline predisposition in these cases. During the screening of 75 CLL cases for kappa and lambda light-chain rearrangements, we could detect a single case with CLL cells expressing two distinct kappa and lambda light chains paired with two separate immunoglobulin heavy-chain variable regions. Furthermore, this patient also developed a prostate carcinoma. Targeted genome sequencing of highly purified light-chain specific CLL clones from this patient and from the prostate carcinoma revealed the presence of a rare germline polymorphism in the POLE gene. Hence, our data suggest that the detected SNP may predispose for cancer, particularly for CLL.

## 1. Introduction

Chronic lymphocytic leukemia (CLL) is a very frequent B cell malignancy of the elderly characterized by the accumulation of mature CD5/CD19 double-positive B cells in peripheral blood and lymphoid organs. Although novel therapies have dramatically improved the treatment outcome of patients, CLL is still considered incurable [1,2]. Typically, CLL is characterized by the expression of a single, monoclonal B cell receptor, consisting of one productive VDJ rearranged immunoglobulin heavy chain (IGH) and a clonal kappa or lambda light chain (IGL-k, IGL-l). In this monoclonal situation, the mutation status of the IGH variable region (IGHV) is a strong prognostic marker, with patients having unmutated IGHV normally experiencing a worse clinical outcome [2]. In a small group of patients as well as in a mouse model for CLL [3], the CLL cells were found to be oligoclonal, carrying multiple (mostly two) productive IGHV rearrangements or dual IGL-k/l expression on the same clone, likely descending from a lack of allelic immune globulin exclusion in B cells [4,5]. In even rarer cases, patients were found to have two distinct CLL clones, one carrying IGL-k and another carrying IGL-l chains, each combined with a different IGHV rearrangement, which are considered to originate separately from two distinct B cells [6,7]. This raises questions of CLL originating from states of clonal hematopoiesis [8] or germline predispositions [9]. Hence, a thorough genetic analysis—which has not been performed in previous studies—of rare CLL patients with two IGHV clones carrying also separate IGL chains may yield important insight into genetic factors predisposing for B cell transformation and CLL development. In the present study, we report on such a rare patient with biclonal CLL and with an additional prostate carcinoma. We performed a comprehensive analysis of the IGHV gene rearrangements and somatic gene mutations of the two separate IGL-k/l clones and the prostate carcinoma cells to get detailed insight into the underlying molecular events causative for dual CLL and prostate cancer development. Our analysis revealed that neither the two separate CLL clones nor a CLL and prostate sample shared a single somatic mutation. However, we noticed the presence of a hemizygous 25 bp germline deletion at a splice site of the POLE gene, which encodes the replicative polymerase epsilon. This specific deletion is annotated in the NCBI dbSNP database as single nucleotide polymorphism (SNP), which occurs at very low incidence yet has no assigned clinical relevance [10]. Our data propose that the detected POLE SNP predisposes to the development of cancer, particularly CLL.

## 2. Results

**Figure 1:**
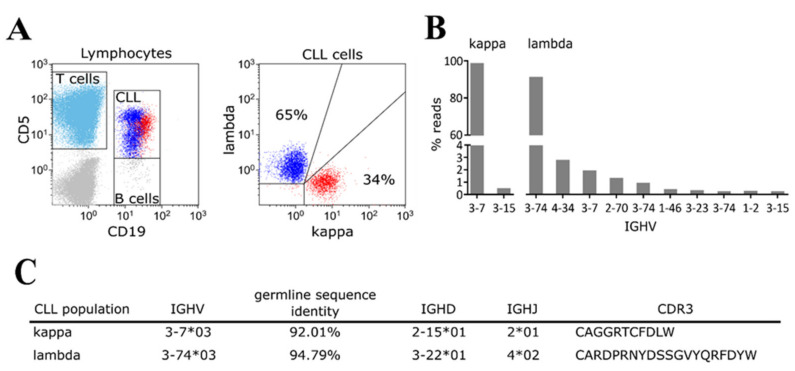
Flow cytometric staining and B cell receptor analysis of a CLL patient with dual clones. (**A**) Refractory CLL with two distinct immunoglobulin light chain clones (kappa/lambda) was measured in flow cytometry 27 months after treatment initiation with ibrutinib. (**B**) Analysis of B cell receptors of sorted kappa and lambda CLL cells taken 18 months prior to ibrutinib therapy revealed two distinct major IGHV clones in kappa and lambda light chains. (**C**) Overview of both major IGH clones found in the sorted kappa and lambda light-chain CLL cells. Both showed mutated (92.01% and 94.79% germline sequence identity) IGHV regions and distinct IGHD, IGHJ, and CD3 regions.

From the initially screened cohort of 75 CLL patients (Table S1), we detected one patient (ID262) with refractory disease 27 months post obinutuzmab/ibrutinib combination therapy initiation, with a substantial leukemic cell load in peripheral blood with CLL cells expressing IGL-k as well as IGL-l chains (Figure 1A). Initially, the patient was diagnosed with CLL Rai stage 0 at age 62, and subsequently, CLL further progressed to Rai stage III, nine years upon CLL diagnosis. Treatment with an obinutuzumab/ibrutinib combination was initiated in a clinical trial. FISH analysis of the CLL cells two months before obinutuzumab/ibrutinib combination therapy did not reveal any common chromosomal aberrations such as deletions at chromosome 13q, 11q, or 17p or trisomy 12. Staining of thawed biobanked samples of this patient confirmed the presence of dual IGL-k and IGL-l clones already 18 months prior to therapy. Interestingly, this patient was also diagnosed with a prostate carcinoma (pT2c, pN0, M0, Gleason Score 3 + 3) five years post initial CLL diagnosis at age 67; overall, 17 of the 75 monitored CLL patients were diagnosed with further malignancies, as is commonly reported in the literature [11]. From a biobanked peripheral blood sample, we separated the two CLL clones by automated cell sorting based on IGL expression using kappa and lambda specific antibodies and assessed the IGHV gene rearrangements by IGHV leader sequencing of each IGL clone (Figure 1B).

**Figure 2:**
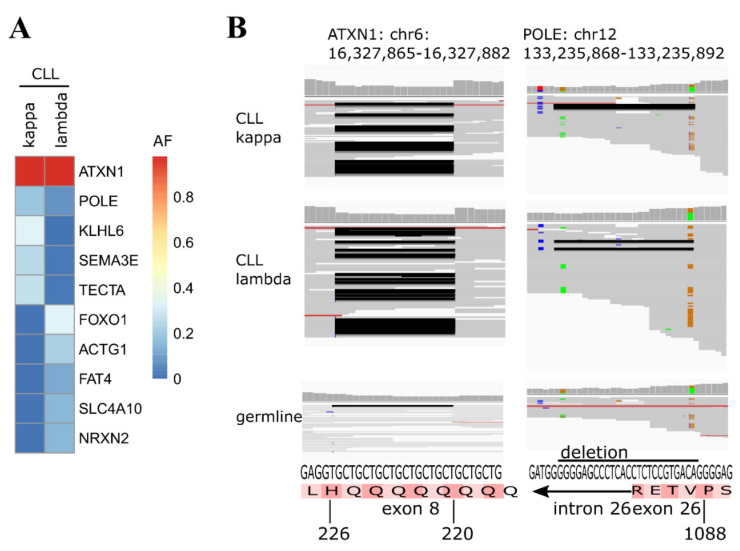
Focused exome sequencing of individually purified CLL kappa and lambda clones and a prostate carcinoma sample. (**A**) Allelic frequencies (AF) of all genes carrying mutations in the prostate carcinoma; the kappa and lambda clones are shown. All samples share the same mutations in the ATXN1 and POLE genes. (**B**) In-frame deletion within the ATXN1 gene and 25 nucleotide splice site within the POLE1 gene are depicted for all cancer samples and the corresponding germline control from a buccal swab.

Our analysis revealed that the kappa and lambda clones in this sample had different IGHV gene usage, with the kappa clone being IGHV 3-7*03 and the lambda clone being IGHV 3-74*03 and distinct IGHD, IGHJ, and CDR3 sequences. The IGHV chains had a sequence homology to the germline IGHV sequences of 95% and 92%, respectively, and therefore, they are classified as mutated IGHV (Figure 1C). The sequence analysis further revealed only one predominant productive rearrangement for either clone, excluding the possibility that the dual CLL originated from a secondary rearrangement of the second IGH/L alleles within the initial CLL clone (Figure 1B). Subsequently, we subjected the two CLL clones and a sample from the resected prostate carcinoma to a focused exome sequencing, covering 6110 disease-related target genes, and identified individual somatic mutations by comparison to genomic DNA isolated from buccal swabs. The focused exome sequencing revealed specific mutations for kappa and lambda clones, comprising mutations in known cancer genes such as FAT4, FOXO1 (IGL-l clone) [12], and the known CLL driver gene KHLH6 (IGL-k clone) [13] (Figure 2A). In the prostate carcinoma sample (tumor cell content of the analyzed FFPE section was determined >95% in the additional microdissection of prostatic carcinoma infiltrated areas; a microscopic visualization showed no CLL infiltrates), we detected two mutations, which we also found in the two CLL clones: an in-frame p220–226 deletion within the ATXN1 gene and a 25 nucleotide splice site deletion within the POLE gene, leading to impaired splicing of intron 26 (Figure 2).

**Figure 3:**
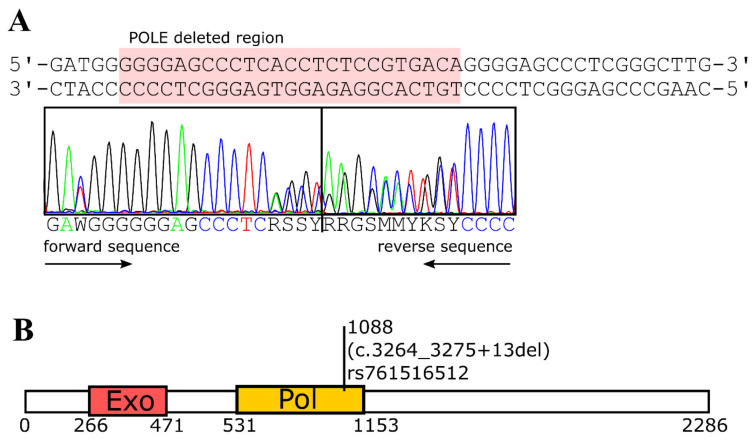
Sanger sequencing of POLE. (**A**) Sequencing shows the presence of POLE mutation in the germline corresponding to SNP rs761516512. (**B**) Schematic representation of POLE, indicating exonuclease (Exo) and polymerase (Pol) domains and the respective amino acid positions.

As coverage for these two positions was very low in the germline sample (135 reads mean coverage versus 22 or 40 reads coverage for POLE or ATXN1) and as both deletions were annotated as polymorphisms in the SNP database [14], we performed Sanger sequencing to check for the presence of these deletions in the cancer and germline samples. Indeed, Sanger sequencing revealed that both deletions were also present in the germline sample (Figure 3 and Supplementary Figure S1). While the observed ATXN1 polymorphism comprises a very frequent length polymorphism of a poly-Q repeat with no putative functional impact as long as the repeat size does not exceed 32 amino acid residues [15], the POLE SNP (rs761516512, c.3264_3275 + 13del) is reported to be extremely rare among the population, with an incidence of 0.002% and unknown clinical significance [10]. This variant is a gross deletion of the genomic region encompassing part of exon 26 of the POLE gene, including the exon 26–intron 26 boundary. This creates a premature translational stop signal and is expected to result in a truncated and dysfunctional protein product (Figure 3B).

## 3. Discussion

In this study, we present an in-depth molecular analysis of a unique case with two independent CLL clones and a prostate carcinoma. Although previous investigations already suggested an origin from two independent B cells, thorough genetic evidence for this assumption was missing. We found that all three cancers and particularly the two CLL clones had no shared mutations, which confirms that the two CLL clones likely originated from two different B cells and not from a common B cell precursor with a distinct profile of somatic mutations. In contrast, we describe the presence of a hemizygous POLE deletion already present in the germline DNA of the patient, which impedes correct splicing and thus likely results in translation of a dysfunctional polymerase epsilon protein.

Although we cannot exclude the presence of mutations not covered by our 6110 gene exome panel or of additional unfavorable SNPs that remained undetected in our study, our data suggest that POLE SNP rs761516512 predisposes to a higher incidence for cancer development. Although POLE mutations are not recognized as known driver genes in CLL, the gene is listed as a cancer driver in the cosmic database [12], and germline mutations in exonuclease domains of POLE were shown to be associated with increased risk for colorectal cancer [16]. POLE encodes the replicative DNA polymerase epsilon, and the variant discerned in our study involves a deletion at the splice site at the intron 26–exon 26 border (c.3264_3275 + 13del). This likely creates a premature translational stop signal and is expected to result in an absent or disrupted protein product. This variant is present in population databases at a very low frequency of 0.002% [10], and up to now, no clinical relevance has been assigned to this variant. Previously, missense variants that affect the exonuclease domain of POLE, responsible for proof-reading activity, have been associated with an increased risk for colon cancer [17,18]. In contrast, monoallelic loss-of-function variants, which result in an absent or non-functional truncated POLE protein, were classified as variants of uncertain significance [19]. Our data now point to a role of this SNP in cancer by yet unknown mechanism. It is conceivable that POLE is haploinsufficient for correct replication, or alternatively, the variant translates into a truncated protein that impacts the regulation, assembly, processivity, or fidelity of the replication machinery.

## 4. Materials and Methods

### 4.1. Subjects

We initially monitored a cohort of 75 CLL patients (Table S1) during or after therapy to assess minimal residual disease, using a flow cytometry panel according to previously published methods [20]. The panel includes antibodies specific for CD5, CD19, and immunoglobulin lambda and kappa light chains. The patients gave written informed consent to study participation and data publication (Ethics statement/Ethics number: 415-E/1287/18-2018, Version 5 from the Province of Salzburg) in accordance with the Declaration of Helsinki.

### 4.2. Flow Cytometric Kappa/Lambda Staining and Cell Sorting

To determine the amount of IGL-k and IGL-l positive cells in CLL samples, we performed a flow cytometric staining specific for these two clones in combination with CLL and T cell detection of thawed biobanked and density gradient purified PBMCs. After thawing and washing of a PBMC sample, about 20 million cells were resuspended in 1 mL of PBS supplemented with 0.1% BSA. We added 20 µL anti-lambda-FITC (BD Biosciences, San Jose, CA, USA; clone 1-155-2; cat. 347247), 20 µL anti-kappa-PE (BD Biosciences; clone TB28-2; cat. 347246), 15 µL anti-CD5-PC5 (Beckman Coulter, Brea, CA, USA; clone BL1a; cat. IM2637U), and 15 µL anti CD19-PC7 (Beckman Coulter, clone J3-119, cat IM3628U) antibodies. The cells were incubated with the antibodies for 25 min at 4 °C. After washing, filtering, and resuspending of the cells in RPMI 1640 (*w*/*o* pheno red; Life Technologies, Carlsbad, CA, USA; cat 32404-014) supplemented with 2% fetal calf serum, they were analyzed on a Cytomix FC500 flow cytometer (Beckman Coulter) or sorted on a FACS Aria III (BD Biosciences) for kappa or lambda-positive CLL cell populations (>400,000 cells per population). Measurements and sorts were evaluated with Kaluza Analysis Software 2.1. (Beckman Coulter) or FACS Diva Software 8.0.1 (BD Biosciences).

### 4.3. IGHV Mutation/Rearrangement Analysis

The sorted kappa and lambda-positive CLL populations were further processed for NGS-based IGHV mutation analysis by LymphoTrack^®^ Dx IGHV Leader Somatic Hypermutation Assay Panel—MiSeq^®^ (Invivoscribe, San Diego, CA, USA; cat 91210069) according to the manufacturer’s recommendation with minor changes. In short, we extracted DNA from the sorted populations according to Qiagen’s protocol of the DNeasy Blood & Tissue Kit (Qiagen, Venlo, Netherlands; cat 69506). We prediluted the extracted DNA to about 10 ng/µL and used 2.5 µL for further processing. We added 22.5 µL IGHV leader master mix (Invivoscribe) and 0.1 µL Eagle Taq DNA polymerase (Roche, Basel, Switzerland; cat 05206944190) to a final volume of 25.1 µL. We used the PCR program according to the LymphoTrack^®^ Dx protocol and a Biometra T3000 thermocycler (Analytic Jena, Jena, Germany). Further PCR product cleanup was performed starting with 25 µL of AMPure XP reagent (Beckman Coulter, cat. A63881) per cleanup and further steps according to the LymphoTrack^®^ Dx protocol. We eluted each sample with 15 µL of 10 mM Tris solution with pH 8.0. After PCR product check with the 4200 TapeStation analyzer and D1000 screen tapes (Agilent, Santa Clara, CA, USA), the concentration was determined with Qubit and a dsDNA HS Assay Kit (ThermoFisher, Waltham, MA, USA). Further processing strictly followed the LymphoTrack Dx protocol. Then, 15 pM final NGS library were sequenced with the MiSeq reagent kit v3 and a 2x 301 bp read length on a MiSeq Sequencer (Illumina, San Diega, CA, USA). Analysis of the sequencing runs was performed using LymphoTrack Software (Invivoscribe). The obtained sequences were further subjected to IMGT/V-QEST [21,22] and ARResT/AssignSubsets [23] for detailed analysis.

### 4.4. Focused Exome Sequencing

To analyze 6110 disease-associated genes in the kappa or lambda-sorted CLL samples, the prostate cancer FFPE sample from radical prostatectomy, and the buccal swab sample for germline analysis, we used the Agilent SureSelect QXT focused exome reagent kit and capture library (Agilent; cat. G9683A, 5190–7787). DNA extraction was performed as described above. Samples were prepared according to Agilent’s SureSelectQXT Target Enrichment protocol for the Illumina paired-end multiplexed sequencing library using the 200 ng DNA input option or the FFPE-optimized option for the prostate specimen, respectively. The prepared library was analyzed on a NextSeq 550 platform with 2x 100 bp read length and a 300 cycle kit v2. As seeding concentration, 1.6 pM was used.

### 4.5. Sequencing Data Analysis

Raw sequencing basecount files were demultiplexed using Illumina bcl2fastq software (v2.19.1.403), and the demultiplexed fastq files were trimmed using Trimmomatic (v0.33, TruSeq3PE_adapters and default settings) [24]. Trimmed paired fastq files were aligned to hg19 reference genome by bwa mem (v0.7.12-r1039, arXiv:1303.3997), and the aligned bam files were processed using the following tools: picard MarkDuplicates (v2.22.4, Broad Institute), GATK RealignerTargetCreator (v3.7-0) [25], GATK IndelRealigner (v3.7-0, maxReads 1000000), GATK BaseRecalibrator (v4.1.7.0, known sites hg19.dbsnp151.common_all_20180423, S07084713_SureSelect_Focused_Exome_Human/S07084713_Padded.bed), and GATK ApplyBQSR (v4.1.7.0). Processed bam files were used to generate mpileup files (buccal swap vs. each tumor sample) by samtools mpileup (v1.5, -B –q 1) [26]. We used VarScan2 (v2.4.4) [27] and the pipeline proposed by Koboldt et al. [28] for variant calling with the following commands and options: somatic –min-var-freq 0.05, processSomatic –min-tumor-freq 0.02 –max-normal-freq 0.05 *p*-value 0.05, somaticFilter. Bam-readcount (v0.8.0) [29] was used to calculate the read depth at high confidence variant sites and VarScan2 fpfilter (–dream3-settings 1 –min-var-avgrl 70 –min-ref-avgrl 70) produced high-stringency filtered variants. Finally, variants with a somatic score of 30 or higher were annotated by ANNOVAR (v2017-07-17) [30] and the following databases: refGene, avsnp150, clinvar_20200316, cosmic91, popfreq_all_20150413. All variants were checked for artifacts in the Integrative Genomics Viewer (IGV) [31].

### 4.6. Sanger Sequencing

For confirmation of POLE and ATX1 aberrations, we used DNA obtained as described above from the sorted kappa and lambda clone CLL cells, the prostate cancer FFPE sample from radical prostatectomy, and the buccal swab sample. Primer sequences for POLE were TAAACACAGCCCAAATCTGTAAGGAACC (forward), GGAGCAGAAGTCTACGTCCATCAGCAC (reverse), and for ATXN1, they were GGAAATGTGGACGTACTGGTTCTGCTG (forward), CTATTCCACTCTGCTGGCCAACATGG (reverse). For PCR amplification, we prepared PCR reactions consisting of 10 µL of 2x Phusion Master Mix with HF buffer (ThermoFisher, cat. F531S), 1 µL of each forward and reverse primer diluted to 10 pmol/µL, 20 ng DNA, and added nuclease-free water to a total reaction volume of 20 µL. For POLE, we used the following temperature gradient: (1) 98 °C 30 s, (2) 98 °C 10 s, (3) 63 °C 5 s, (4) 72 °C 20 s, go to step 2 30 times, (5) 72 °C 10 min, (6) 4 °C hold. For ATXN1, we used the following conditions: (1) 98 °C 30 s, (2) 98 °C 10 s, (3) 72 °C 20 s, go to step 2 30 times, (4) 72 °C 10 min, 4 °C hold. All reactions were performed on a Biometra Trio (Analytic Jena, Jena, Germany). After PCR amplification, 5 µL of all PCR products were cleaned up with 2 µL ExoSAP-ITTM solution (ThermoFisher; cat. 78200.200.UL) at 37 °C for 15 min, 80 °C for 15 min, 4 °C hold and prepared for Sanger sequencing by adding 2 µL cleaned up PCR product to 2 µL 5x Cycle Sequencing buffer, 1 µL BigDyeTM Terminator v3.1(ThermoFisher, BigDyeTM Terminator v3.1 Cycle Sequencing Kit, cat 4337454), and 0.5 µL forward or reverse primer. The reactions were filled to a final volume of 10 µL with nuclease-free water. The cycle sequencing program was as follows: (1) 96 °C 1 min, (2) 96 °C 10 s, (3) 50 °C 10 s, (4) 60 °C 3 min, go to step 2 31 times, 4 °C hold. Before subjecting the samples to Sanger sequencing, a XTerminatorTM purification (ThermoFisher, BigDye XTerminatorTM Purification Kit, cat 4376486) was performed. To each 10 µL sample, 40 µL SAMTM solution and 6 µL XTerminatorTM solution were added and shook for 30 min at 1800 rpm on a ThermoMixer (Eppendorf, Hamburg, Germany), centrifuged 2 min at 1000× *g* and analyzed on a 3500 Series 8-capillary Genetic Analyzer (ThermoFisher) with POP-7TM polymer separation matrix (ThermoFisher). Sequences were evaluated using Chromas Lite Software 2.01 (Technelysium, South Brisbane, Australia).

## Data Availability

All primary data are available upon reasonable request.

## Supplementary Materials

The following are available online at https://www.mdpi.com/article/10.3390/ijms22179410/s1, Supplementary Table S1: Patient characteristics of 75 CLL patients screened in this study.
