# Supplementary material for: A POLE Splice Site Deletion Detected in a Patient with Biclonal CLL and Prostate Cancer: A Case Report
Source: Int J Mol Sci. 2021 Aug 30;22(17):9410. doi: 10.3390/ijms22179410 (PMC8431722; doi:10.3390/ijms22179410)
Supplement: Supplementary file 1 [file ijms-22-09410-s001.zip › Figure S1.pdf]

**Figure S1**

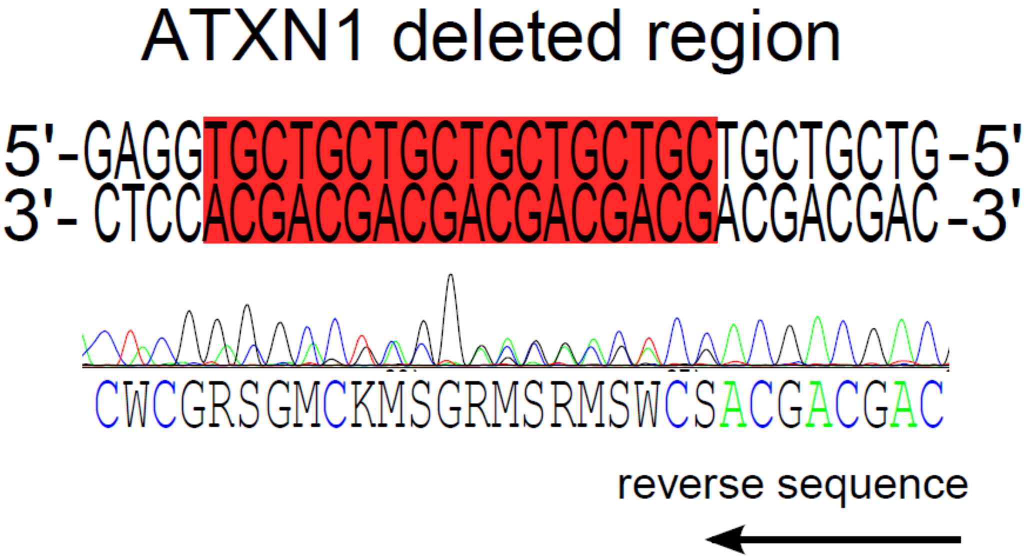


**Supplementary Figure S1:** Sanger sequencing on ATXN1 PCR-amplified from buccal swab DNA confirms presence of hemizygous length polymorphism in germline DNA.
