# Supplementary material for: A POLE Splice Site Deletion Detected in a Patient with Biclonal CLL and Prostate Cancer: A Case Report
Source: Int J Mol Sci. 2021 Aug 30;22(17):9410. doi: 10.3390/ijms22179410 (PMC8431722; doi:10.3390/ijms22179410)
Supplement: Supplementary file 1 [file ijms-22-09410-s001.zip › Table S1.pdf]

patID	age	sex	secondary_malignancy	most recent therapy prior sampling	previous therapies
5	67.0	M	polycythemia	Benda-R	
25	61.0	M	polycythemia	Revlirit	FCR, Benda-R, Rituximab
36	68.0	F		Benda-R	Chlorambucil, Flusalem, Benda-R
61	60.0	M	follicular thyroid carcinoma	Lenalidomide	Rituximab
67	62.0	F		Venetoclax	FCR
122	86.0	F		untreated	
186	77.0	F	mantle cell lymphoma	FCR	
187	72.0	M		Venetoclax + Rituximab	FCR, Gupta, Ibrutinib
262	73.0	M	prostate cancer	Ibrutinib + Obinutuzumab	
283	68.0	M		Rituximab	Revlirit, Benda-R
293	85.0	F	lung cancer	Ibrutinib + Obinutuzumab	
298	80.0	F		RevliRit	
299	62.0	M	lung cancer	Venetoclax	FCR
337	76.0	M		Rituximab	FCR, Benda-R
341	82.0	F		Bendamustin + Obinutuzumab + Dexamethason	FCR, Gupta, Benda-R
359	61.0	M		Venetoclax	Revlirit, Ibrutinib
435	71.0	F	breast cancer	FCR	
449	68.0	F		Benda-R	
472	83.0	F		Benda-R	
491	79.0	M		Ibrutinib	Alemtuzumab, Ofatumumab
496	79.0	M		Rituximab	Revlirit
501	74.0	F		Rituximab	FCR
503	55.0	M		FCR	
531	81.0	M	prostate cancer	Benda-R	CR
557	64.0	M		Ibrutinib	FCR, Rituximab, Obinutuzumab
563	77.0	M		Benda-R	
565	67.0	M	prostate cancer	Rituximab	Benda-R
574	76.0	M		Benda-R	
590	72.0	M	MDS	Venetoclax	Benda-R, FCR, Ofatumumab, Ibrutinib
594	69.0	F		Rituximab + Venetoclax	Revlirit, Rituximab, Idelalisib, Ibrutinib
608	75.0	F		Rituximab + Venetoclax	Benda-R
609	78.0	F		Benda-R	
610	63.0	M		FCR	
613	74.0	M		Benda-R	
618	85.0	F		Benda-R	Gupta
621	73.0	M	kidney cancer	Benda-R	
633	66.0	F		RevliRit	
641	64.0	M		Rituximab	Benda-R
642	79.0	F		Rituximab	Benda-R
644	83.0	M		Ibrutinib	Chlorambucil
648	78.0	M		untreated	
662	71.0	M	colon cancer	Benda-R	
679	57.0	M	prostate cancer	FCR	
690	90.0	F		Ibrutinib	Benda-R

695	53.0	M		FCR	
696	70.0	M		Benda-R	
702	86.0	F		untreated	
706	64.0	F		Benda-R	FCR
749	59.0	M		Ibrutinib	Gupta
777	63.0	M		Benda-R	
780	69.0	M		Benda-R	CR, Rituximab, Gupta
781	83.0	F		Chlorambucil + Obinutuzumab	
790	73.0	F		Benda-R	
796	55.0	F		Benda-R	
817	86.0	M	kidney cancer	Benda-R	
824	49.0	F		Rituximab, Idelalisib	
857	77.0	M	melanoma	Acalabrutinib	Benda-R, FCR, Rituximab
863	57.0	F		Rituximab	FCR
869	77.0	M		Benda-R	
877	79.0	M		Rituximab	Benda-R
895	77.0	M		Benda-R	
901	73.0	F		Benda-R	
914	58.0	M		FCR	
916	73.0	M		Benda-R	
933	73.0	M	prostate cancer	Benda-R	
962	67.0	F		Benda-R	
968	80.0	F		Bendamustin	
981	73.0	M		Benda-R	
1004	68.0	F		Benda-R	
1006	76.0	M		Benda-R	
1028	63.0	F		FCR	
1040	52.0	M		FCR	
1051	80.0	F		Benda-R	
1072	68.0	M		Benda-R	
1074	63.0	M		FCR	
